# Clinicopathologic features of two unrelated autopsied patients with Charcot-Marie-Tooth disease carrying *MFN2* gene mutation

**DOI:** 10.1186/s40478-023-01692-w

**Published:** 2023-12-20

**Authors:** Hideki Hayashi, Rie Saito, Hidetomo Tanaka, Norikazu Hara, Shin Koide, Yosuke Yonemochi, Tetsuo Ozawa, Mariko Hokari, Yasuko Toyoshima, Akinori Miyashita, Osamu Onodera, Kouichirou Okamoto, Takeshi Ikeuchi, Takashi Nakajima, Akiyoshi Kakita

**Affiliations:** 1 Department of Pathology, Brain Research Institute, Niigata University, 1-757 Asahimachi, Chuo-Ku, Niigata, 951-8585 Japan; 2 Department of Neurology, Brain Research Institute, Niigata University, 1-757 Asahimachi, Chuo-Ku, Niigata, 951-8585 Japan; 3 Department of Molecular Genetics, Brain Research Institute, Niigata University, 1-757 Asahimachi, Chuo-Ku, Niigata, 951-8585 Japan; 4 Departments of Neurology, National Hospital Organization Niigata National Hospital, 3-52 Akasakachou, Kashiwazaki, 945-8585 Japan; 5 Department of Internal Medicine, National Hospital Organization Niigata National Hospital, 3-52 Akasakachou, Kashiwazaki, 945-8585 Japan; 6 Department of Neurology, Niigata City General Hospital, 463-7 Shumoku, Chuo-Ku, Niigata, 950-1197 Japan; 7 Department of Neurology, Brain Disease Center, Agano Hospital, 6317-15 Yasuda, Agano, Niigata, 959-2221 Japan; 8 Department of Translational Research, Brain Research Institute, Niigata University, 1-757 Asahimachi, Chuo-Ku, Niigata, 951-8585 Japan

To the editor

Charcot-Marie-Tooth disease type 2A2 (CMT2A2) is the second most common type of CMT, characterized by axonal neuropathy, and 9–20% of affected patients present with optic atrophy [6, 8]. It is caused by mutations in the *mitofusin2* (*MFN2*), which encodes a component of the outer mitochondrial membrane, and is essential for mitochondrial fusion [2]. Although a number of clinical studies have been reported, the neuropathologic features remain unknown. Here we evaluated the clinicopathologic features of two unrelated autopsied patients harboring *MFN2* mutation.

The two unrelated males (patients 1 and 2) without similar symptoms in their families exhibited similar clinical characteristics, having developed gait disturbance in early childhood, followed by gradually progressive muscle atrophy and sensory disturbance in the upper and lower extremities. No formal ophthalmologic examination was performed and no subjective symptoms of visual impairment were noted. In both patients, MRI imaging demonstrated atrophy of the optic pathway and FLAIR hyperintensities in the subcortical white matter, extending from the middle cerebellar peduncles (MCPs) to the cerebellar white matter (WM), being accompanied by enlarged lateral ventricles in patient 1 (Fig. 1a–e). Both patients died in their 70 s, and at autopsy the brains weighed 1250 g and 1100 g, respectively. The brain weight of patient 1 was within the normal range, whereas that of patient 2 was moderately reduced due to old cerebral hemorrhage. The patients’ clinical features are summarized in Table 1. Genetic analysis revealed a heterozygous missense mutation, p.Arg364Trp (c.1090 C > T), in *MFN2* in both patients (Fig. 1f).

**Fig. 1:**
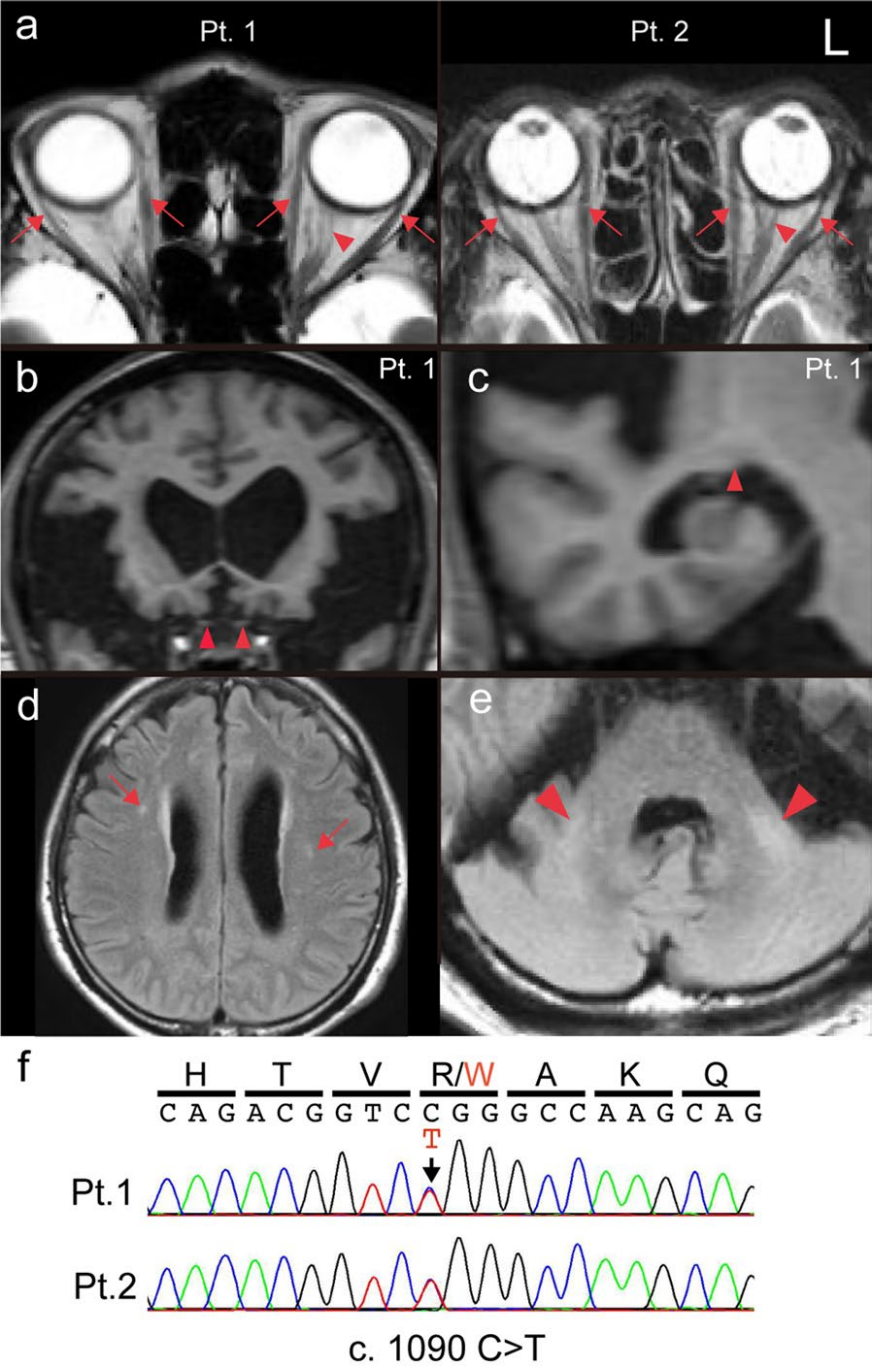
MRI findings and genetic analysis. **a** Brain MRI images of patients 1 and 2 (Pt. 1 and 2). The T2-weighted images were acquired at the age of 69 (Pt. 1) and 50 years (Pt. 2). Hyperintensity in the intraorbital optic nerves (Pt. 1 and 2, *red arrowheads*) and preserved extraocular muscles (Pt. 1 and 2, *red arrows*) are evident. Coronal and sagittal T1-weighted images of Pt. 1 show atrophy of **b** the intracranial optic nerves (*red arrowheads*) and **c** lateral geniculate body (*red arrowhead*). Note the presence of disproportionately enlarged subarachnoid space hydrocephalus (DESH), a hallmark of idiopathic normal pressure hydrocephalus with enlarged lateral ventricles. Hyperintensities in **d** the subcortical white matter (Pt. 1, *red arrows*) and **e** extending from the middle cerebellar peduncles (Pt. 1, *red arrowheads*) to the lateral side of the cerebellar white matter. **f** Sanger sequencing of the mutation in *MFN2*. *Red arrow* indicates the mutation. FLAIR, fluid attenuated inversion recovery; Pt, patient; L: Left side of the brain

**Table 1:** Clinical features of patients with *MFN2* mutation

	Patient 1	Patient 2
Age at time of death, years (y)/ sex	72 y/ Male	72 y/ Male
Consanguinity	–	+
Family history	–	–
Clinical symptoms		
Age at onset, y	5 y	2 y
Initial symptom	Gait instability	Gait instability
Muscle atrophy (U/L)*	+ , distal/ + , distal	+ , distal/ + , distal
Proximal muscle weakness (MMT: U/L)	Moderate (3–5/1–4)	Moderate (3–4/0–3)
Distal muscle weakness (MMT: U/L)	Severe (1/0)	Severe (1/0)
Deep tendon reflexes (U/L)*	Loss/loss	Loss/loss
Sensory impairment* (superficial/deep)	+ / +	+ / N.A.
Foot deformities	+	+
Visual impairment	N.A.	N.A.
Hearing loss	–	–
Scoliosis	–	–
Mental retardation	–	–
Cognitive decline	–	–
Cause of death	Aspiration pneumonia	Renal abscess
MRI findings	Optic tract** atrophy, enlarged lateral ventricle, DESH, subcortical WM and MCP to cerebellar WM hyperintensities on FLAIR	Optic nerve atrophy, Lt. putaminal hemorrhage subcortical WM hyperintensity on FLAIR

+ and –, presence and absence, respectively; *N.A.* Not available. *U* Upper limbs; *L* Lower limbs; *MMT* Manual muscle testing (0: no contraction; 1: flickering contraction; 2: full range of motion with eliminated gravity, 3: full range of motion against gravity, 4: full range of motion against gravity with minimal resistance, 5: full range of motion against gravity with maximal resistance); *LGB* Lateral geniculate body; *WM* White matter; *DESH* Disproportionately enlarged subarachnoid space hydrocephalus; *MCP* middle cerebellar peduncle. *Neurological examinations of patients 1 and 2 were performed at the ages of 57 and 39 years, respectively. **Optic nerve and LGB

The histopathologic features of the nervous system in patients 1 and 2 were quite similar, being characterized by degeneration of the visual, dorsal column pathway, and dorsal spinocerebellar and corticospinal tracts (Table 2). These changes were more severe in patient 1. In the visual pathway, optic nerves exhibited atrophic change (Fig. 2a) with axonal depletion and swelling (Fig. 2b and c). Severe neuronal loss in the lateral geniculate body (LGB) (Fig. 2d) and moderate loss of large neurons in layers IV of the striate cortex (Fig. 2e) were also evident. Unfortunately, we were unable to assess the LGB in patient 2 due to putaminal hemorrhage (Additional file 1: Fig. S1). In the dorsal column pathway, the dorsal column, especially in the gracile fasciculus, showed severe atrophy and myelin pallor (Fig. 2f), accompanied by severe neuronal loss in the gracile and cuneate nuclei. Loss of ganglion cells in the dorsal root ganglion was also observed (Fig. 2g). The dorsal spinocerebellar tract showed myelin pallor with severe neuronal loss in Clarke's nucleus (Fig. 2h), while the cerebellar cortex was preserved. In the motor system, the lumbar anterior horn cells showed severe neuronal loss (Fig. 2i), and the iliopsoas muscle exhibited neurogenic changes (Fig. 2j). In the spinal anterior horns of both patients, pTDP-43 immunostaining revealed no granular, round, or skein-like neuronal inclusions or glial cytoplasmic inclusions usually observed in sporadic amyotrophic lateral sclerosis (ALS). On the other hand, ubiquitin immunostaining revealed a few neurons with diffuse positive structures in the cytoplasm and neurites only in patient 1 (Additional file 1: Fig. S3). The significance of the ubiquitin-positive structures was unclear, but they did not exhibit the common immunohistochemical staining patterns of ubiquitin observed in sporadic ALS. Further neuropathologic studies of CMT2A2 will be necessary to clarify the involvement of the ubiquitin–proteasome system.

**Table 2:** Distribution of neuronal loss with gliosis

	Patient 1	Patient 2
Cerebrum		
Frontal cortex	–	–
Motor cortex	–	–
Temporal cortex	–	–
Parietal cortex	–	–
Occipital cortex (striate cortex)	–/++	–/+
White matter*	–	–
Subcortical area		
mmon (CA1/subiculum)	–/–	–/–
Amygdaloid nucleus	–	–
Caudate nucleus/putamen	–/–	–/ N.A.**
Globus pallidus i/e	–/–	–/–
Thalamus	–	–
Subthalamic nucleus	–	–
Lateral geniculate body	+++	N.A.**
Brainstem		
Substantia nigra	–	–
Pontine nucleus	–	–
Cranial motor nerve nuclei (IV/V motor/VI/VII/XII)	–/–/–/N.A./–	–/–/N.A./–/–
Ambiguus nucleus	–	–
Dorsal vagal nucleus	–	–
Inferior olivary nucleus	–	–
Reticular formation	–	–
Vestibular/cochlear nuclei	–/N.A.	–/N.A.
Gracile/cuneate nuclei	+++/++	+++/++
Cerebellum		
Cortex/white matter*	–/+	–/+
Dentate nucleus	–	–
Spinal cord		
Anterior horn (C/Th/L/S)	+/++/++/++	+/+/++/++
Clarke’s nucleus	+++	+++
Intermediolateral nucleus	–	–
Lateral corticospinal tract*	+	+
Spinocerebellar tract (Vent./Dors.)*	–/+	–/+
Anterior funiculus (C/Th/L/S)*	–/–/–/–	++/+ ± /–
Posterior funiculus (gracile/cuneate)*	++/+	++/+
Peripheral nerve		
Dorsal root ganglion (C/L)	+/++	+/+
Anterior nerve root (C/L)*	+/++	+/N.A.
Posterior nerve root (C/L)*	+/++	+/N.A
Sural nerve*	+++	+++
Sympathetic ganglion	–	–
Other		
Optic nerve*	+++	++

Neuronal loss with gliosis, +++: Severe, ++: Moderate, +: Mild, –: None, *N.A.* Not available. i/e: Internal/external segment; *Vent.* Ventral; *Dors.* Dorsal

*Loss of myelinated fibers. **Not available due to putaminal hemorrhage

**Fig. 2:**
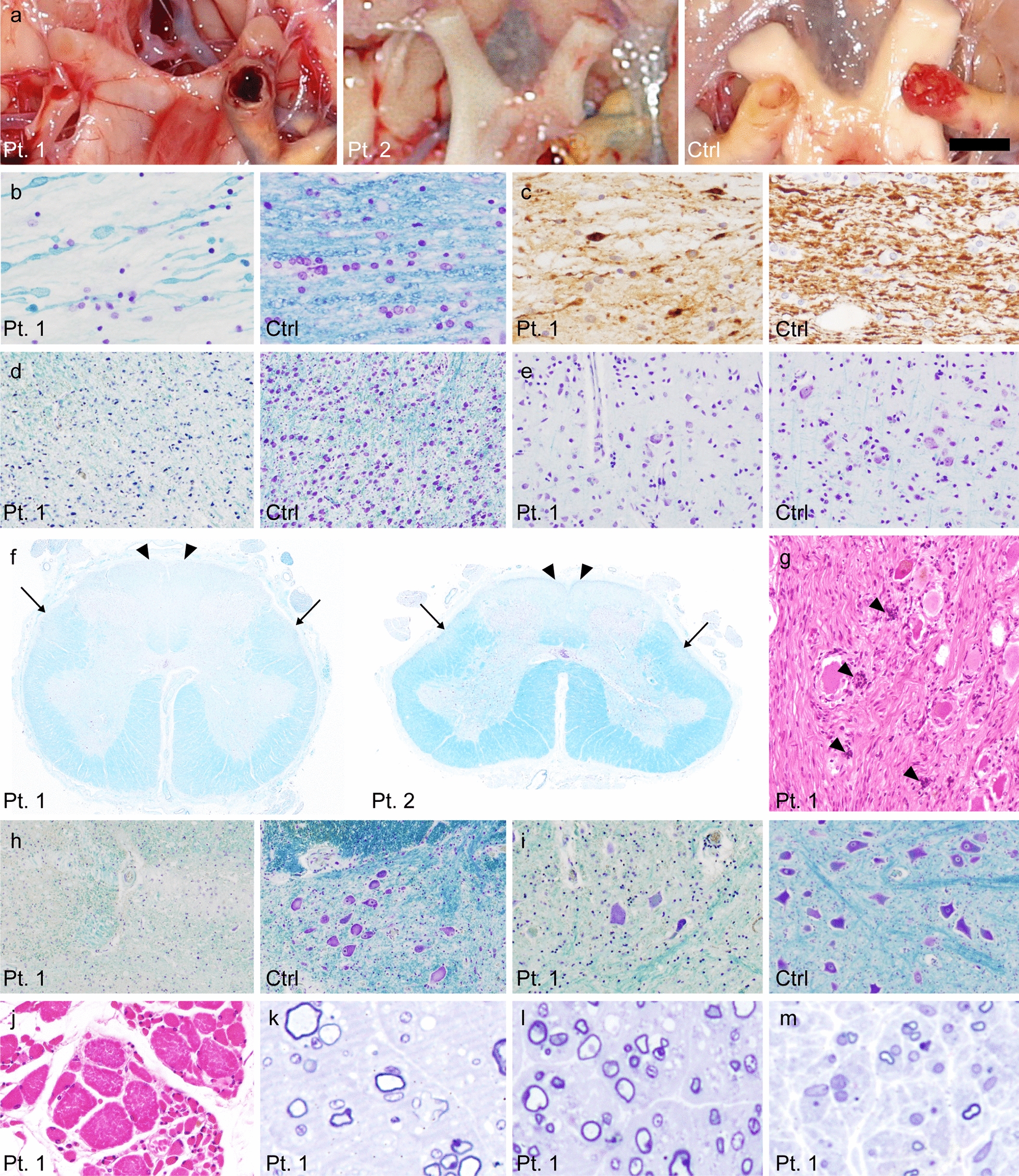
Neuropathologic findings. **a** Macroscopic appearance of the optic nerves. Atrophy is evident. **b** Depletion of myelinated fibers and **c** loss and occasional swelling of axons in the optic nerve. Klüver-Barrera staining (KB) and phosphorylated neurofilament (pNF) immunohistochemistry. **d** Moderate neuronal loss in the lateral geniculate body (LGB) and **e** large neurons in layer IV of the striate cortex. KB staining. **f** Atrophy and myelin pallor of the lateral corticospinal and dorsal spinocerebellar tracts (*arrows*), and dorsal columns (*arrowheads*). KB staining. **g** Loss of ganglion cells with a Nageotte nodule (*arrowheads*) in the dorsal root ganglion of the lumbar spinal cord. HE staining. **h** Severe neuronal loss in Clarke’s nucleus and **i** the anterior horn. KB staining. **j** Neurogenic change with small grouped atrophy is evident in the iliopsoas muscle **k**–**m** Coronal sections of the **k** anterior and **l** posterior nerve roots of the lumbar cord and **m** sural nerve. Severe loss of large and small myelinated fibers and thinning myelin sheath of residual fibers. Toluidine blue staining. Ctrl, control; Pt, patient. Bar in **a** = 5 mm in **a**; 1.3 mm in **f**; 200 μm in **d**, **i**; 160 μm in **h**, **j**; 100 μm in **e**, **g**; 50 μm in **b**, **c**, **j**; 30 μm in **k**, **l**, **m**

On the other hand, the primary motor area was not affected despite mild degeneration in the lateral corticospinal tract (Additional file 1: Fig. S2 and Fig. 2f). Histological observation of the lesions showing FLAIR-hyperintense lesions in the subcortical white matter and MCPs on MRI, which have been reported in CMT2A2 [3, 4], was not possible, but cerebellar white matter possibly associated with the hyperintensity showed mild myelin pallor (Additional file 1: Fig. S2). Other cerebral and brainstem nuclei were almost unaffected in both patients (data not shown).

In the peripheral nerves, atrophy of the anterior and posterior roots was evident, histopathologically showing severe loss of thick and thin myelinated axons and thinning of the remaining myelin sheaths (Fig. 2k–m). The sural nerve was the most severely affected (Fig. 2m). No onion bulb formations were observed (Fig. 2k-m).

Ultrastructurally, longitudinal sections of the sural nerve and the posterior spinal nerve roots demonstrated aberrant aggregated and round mitochondria in the patients (Fig. 3a, a’, c, c’), while normal elongated and thread-like mitochondria were randomly dispersed in the axons of the controls (Fig. 3b, b’). In the optic nerves, similar abnormal mitochondria were scattered (Additional file 1: Fig. S4).

**Fig. 3:**
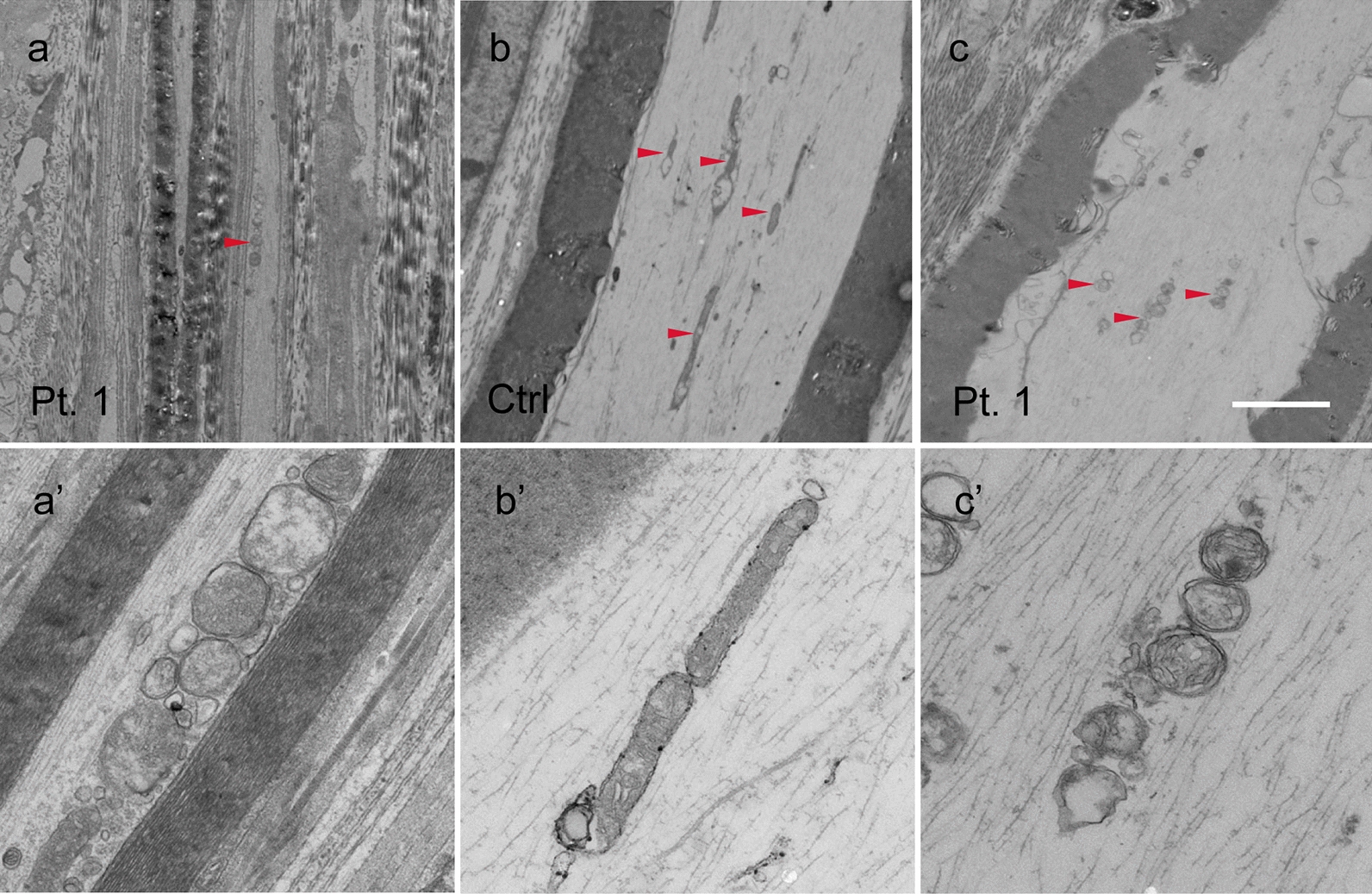
Ultrastructure of mitochondria in the axons of peripheral nerves. Longitudinal section of the sural nerves obtained from **a** patient 1 and **b** a control, and **c** the patient’s posterior root. **a’**–**c’** The magnified images. In the patient, **a** mitochondria are located in the periphery within the axon and **a’** show aberrant aggregation and circularization (*arrowhead*), while **b** mitochondria are randomly distributed within the axon and **b’** appear normally elongated in the control (*arrowheads*). **c**, **c’** In the posterior root, similar abnormal mitochondria are scattered (*arrowheads*). Ctrl, control; Pt, patient. Bar in **c** = 2.5 μm for **a**, **b**, **c**; 500 nm for **a’**, **b’**, **c’**

We have presented the first genetically confirmed autopsy patients with CMT2A2, and have demonstrated that both patients exhibited a similar pathology, showing degeneration in multiple systems in the CNS as well as peripheral nerves and ultrastructural changes in the mitochondria of the peripheral and optic nerves.

In CMT2A2, the p.Arg364Trp identified in our patients is known to be associated with optic atrophy and an early progressive clinical course [7], and this was consistent with the clinicopathologic features of the present patients. Based on our observations, severe muscle atrophy and sensory disturbance including impaired proprioception prominent in the lower limbs [8] would be associated with loss of anterior horn cells and anterior roots, and degeneration of the dorsal column pathway along with Clark’s dorsal nucleus, respectively. We also found that the optic nerves had been markedly affected, followed by degeneration of the LGB and primary visual cortex, especially layer IV where the majority of geniculocortical afferents terminate [1]. All of these affected systems were long pathways, and axons were more affected in the periphery. Interestingly, the neuronal cell bodies to which the axons directly connected also exhibited a similar "proximal–distal gradient" pattern, although the degree of degeneration was milder than that in axons.

Mitochondrial morphometry of the peripheral and optic nerves in our patients demonstrated the characteristic features of biopsied peripheral nerves reported in 6 cases of early-onset CMT2A2 [9]. In all cases, the mitochondria appeared to be small and roundly aggregated, and these findings were confined to the axons, even though various mutations were involved. By contrast, a study of *Drosophila* disease models reported that mitochondria with *MFN2* mutation showed various morphological changes such as hyperfusion and non-fusion, depending on the location of the mutation, and R364W-like mutation was associated with hyperfusion, with large and round mitochondria [5]. These findings suggest that mutations in *MFN2* may cause defects in mitochondrial fusion and fission, reflecting the essential role of *MFN*2. Although the pathomechanisms of neuronal alterations resulting from *MFN2* mutation remain unknown, the varying degrees of degeneration of the central nervous system in relation to the peripheral nerves may provide clues to the pathogenesis of the disease.

In conclusion, degeneration of peripheral nerves and associated tracts, including neurons, with mitochondrial ultrastructural abnormalities was a pathologic characteristic of patients with *MFN2* mutation. Further clinicopathologic and molecular studies are needed to clarify in more detail the selective vulnerability of sensory and motor neurons in CMT2A2 in the context of *MFN2*-related mitochondrial abnormalities.

### Supplementary Information

**Additional file 1.** Methods and additional clinicopathological data. **Fig. S1**. Brain MRI FLAIR image of patient 2. **Fig. S2**. Neuropathologic findings in the cerebellum and motor cortex of patient 1. **Fig. S3**. Staining for ubiquitin and other antigens in spinal anterior horn cells. **Fig. S4**. Ultrastructure of mitochondria in the axons of the optic nerves, and proximal and distal segments of the posterior nerve roots (formalin-fixed samples).

## Data Availability

The datasets used and analysed during the current study available from the corresponding author on reasonable request.

## Abbreviations

ALS: Amyotrophic lateral sclerosis
CMT2A2: Charcot-Marie-Tooth disease type 2A2
FLAIR: Fluid attenuated inversion recovery
MCP: Middle cerebellar peduncle
*MFN2*: *Mitofusin2* Gene
LGB: Lateral geniculate body
