# Supplementary material for: Clinicopathologic features of two unrelated autopsied patients with Charcot-Marie-Tooth disease carrying MFN2 gene mutation
Source: Acta Neuropathol Commun. 2023 Dec 20;11:207. doi: 10.1186/s40478-023-01692-w (PMC10734194; doi:10.1186/s40478-023-01692-w)
Supplement: Supplementary file 1 — Additional file 1. Methods and additional clinicopathological data. Fig. S1. Brain MRI FLAIR image of patient 2. Fig. S2. Neuropathologic findings in the cerebellum and motor cortex of patient 1. Fig. S3. Staining for ubiquitin and other antigens in spinal anterior horn cells. Fig. S4. Ultrastructure of mitochondria in the axons of the optic nerves, and proximal and distal segments of the posterior nerve roots (formalin-fixed samples). [file 40478_2023_1692_MOESM1_ESM.pdf]

Additional file 1

Letter to the Editor

Clinicopathologic features of two unrelated autopsied patients with Charcot-Marie-Tooth disease carrying *MFN2* gene mutation

Hideki Hayashi[1, 2], Rie Saito[1], Hidetomo Tanaka[1], Norikazu Hara[4], Shin Koide[2, 5], Yosuke Yonemochi[5], Tetsuo Ozawa[6], Mariko Hokari[2, 7], Yasuko Toyoshima[1, 8], Akinori Miyashita[4], Osamu Onodera[2], Kouichirou Okamoto[3], Takeshi Ikeuchi[4], Takashi Nakajima[5], Akiyoshi Kakita[1]

Departments of [1]Pathology, [2]Neurology, [3]Translational Research and [4]Molecular Genetics, Brain Research Institute, Niigata University, 1-757 Asahimachi, Chuo-ku, Niigata 951-8585, Japan. Departments of [5]Neurology and [6]Internal Medicine, National Hospital Organization Niigata National Hospital, 3-52 Akasakachou, Kashiwazaki, 945-8585, Japan. [7]Department of Neurology, Niigata City General Hospital, 463-7 Shumoku, Chuo-ku, Niigata 950-1197, Japan. [8]Department of Neurology, Brain Disease Center, Agano Hospital, 6317-15 Yasuda, Agano, Niigata 959-2221, Japan.

Correspondence to: Rie Saito, MD, PhD
Department of Pathology, Brain Research Institute, Niigata University, 1-757 Asahimachi, Chuo-ku, Niigata 951-8585, Japan.
E-mail: riesaito@bri.niigata-u.ac.jp

Supplementary methods
Supplementary references
Supplementary figure 1: Brain MRI FLAIR image of patient 2.
Supplementary figure 2: Neuropathologic findings in the cerebellum and motor cortex of patient 1.
Supplementary figure 3: Staining for ubiquitin and other antigens in spinal anterior horn cells.
Supplementary figure 4: Ultrastructure of mitochondria in the axons of the optic nerves, and proximal and distal segments of the posterior nerve roots (formalin-fixed samples).

Supplementary methods

Histopathologic analysis

The brains and spinal cords were fixed with 10% buffered formalin, and multiple tissue blocks were embedded in paraffin. Histological examination was performed on 4-μm-thick sections stained with hematoxylin and eosin, and also Klüver-Barrera with Luxol fast blue and Nissl stains. In addition, selected sections were immunostained with antibodies against phosphorylated neurofilament H (BioLegend, Dedham, Massachusetts, USA; 1:2000), ubiquitin (MBL, Nagoya, Japan; 1:1000; antigen retrieval: autoclave), TRDBP (Abnova, Taipei city, Taiwan; 1:500; antigen retrieval: autoclave), and phosphorylated TDP-43 (pS409/410; Cosmo Bio Co., Tokyo, Japan; 1:5000; antigen retrieval: autoclave). Antibodies against amyloid β 11-28 (IBL, Gunma, Japan; 1:50; antigen retrieval: formic acid), phosphorylated tau (Fujirebio, Ghent, Belguim; 1:200) and phosphorylated α-synuclein (Wako, Saitama, Japan; 1:1000; antigen retrieval: formic acid) were used to assess senile pathologic changes based on "ABC" score [4] and the fourth consensus report of the DLB Consortium [3]. Bound antibodies were visualized by the peroxidase-polymer-based method using a Histofine Simple Stain MAX-PO kit (Nichirei, Tokyo, Japan) with diaminobenzidine as the chromogen. Immunostained sections were counterstained with hematoxylin. As a result, the brains of both patients showed no pathological features suggestive of complications arising from Alzheimer's disease (ABC score: A0B1C0 and A0B1C0) or Parkinson's disease (Lewy body disease: none). In addition, ventricular enlargement with a profile of rostal midbrain atrophy on mid-sagittal images, suggestive of the hummingbird sign, was observed in patient 1. There was no histopathologic evidence of progressive supranuclear palsy.

To assess the anterior and posterior nerve roots and sural nerves of patients 1 and 2, tissues were fixed with 2.5% glutaraldehyde in 0.125 M cacodylate buffer and embedded in epoxy resin. Semithin sections were then prepared and stained with toluidine blue.

For electron microscopy analysis of mitochondria in the axons, formalin-fixed optic nerves and lumbar posterior nerve roots (proximal and distal segments at 5-cm intervals) of patients 1 and 2 were post-fixed with 1% osmium tetroxide, dehydrated

through a graded ethanol series, and embedded in Epon 812. We also assessed the above-mentioned anterior and posterior nerve roots and sural nerves. For observation of mitochondria, longitudinally sectioned axons are most suitable. However, the spinal roots and sural nerve of patient 2 could not be observed longituidinally, and therefore we did not include these samples in the study. Ultrathin sections of the optic nerves, anterior and posterior nerve roots and sural nerves were then cut and stained with uranyl acetate and lead citrate. Ultrathin sections were examined with a Hitachi H-7100 electron microscope at 75 kV.

We included the following normal controls for optical and electron microscopy observations. To evaluate the pathology of the visual and spinal tracts, three individuals without any pathologic changes in the visual tract (an 85-year-old man with Alzheimer's disease, an 85-year-old woman with amyotrophic lateral sclerosis, and an 81-year-old man with Parkinson’s disease), and three individuals without any neurological disorders (a man aged 69 years and two women aged 62 and 72 years) were used, respectively. For assessment of peripheral nerves, three individuals (an 81-year-old man with Parkinson’s disease, a 71-year-old woman with congenital myopathy and a 71-year-old man with limbic encephalitis) were used. In addition, for analysis of optic nerves, two individuals (a 62-year-old woman without any neurological disorder and a 71-year-old woman with Parkinson’s disease) were used.

Genetic analysis

Patient 1: Genomic DNA was extracted from the fresh-frozen parietal cortex. The extracted genomic DNA was used to prepare an exome library using the SureSelect Human All Exon V6 kit (Agilent Technologies, Santa Clara, CA, USA). The exome library was sequenced on an Illumina NovaSeq 6000 Sequencing System in 151-cycle paired-end mode. The sequenced reads were loaded onto *fastp* version 0.19.5 with default settings for quality control and adapter trimming. The cleaned reads were mapped to the human reference genome hg38 using *BWA-MEM* 0.7.15-r1140 [2] with default settings. The subsequent analyses—read processing, variant calling, and variant filtration—were conducted according to the GATK4 Best Practice recommendations [6]. The resulting variant call sets were annotated using *snpEff* 4.3t [1] and stored in an

SQLite database using *gemini* 0.20.1 [5].

To identify pathogenic variants, we selected variants showing autosomal dominant inheritance, autosomal recessive inheritance, or a compound heterozygous pattern. We filtered out variants lacking protein alterations. For autosomal dominant inheritance, we applied additional filters: a CADD score of <15 and a maximum allele frequency of >0.1% in each ethnic population in the following publicly available databases: gnomAD release 2.1 and 3.0 (https://gnomad.broadinstitute.org/), HGVD version 2.3 (http://www.hgvd.genome.med.kyoto-u.ac.jp/), and jMorp version 14KJPN (https://jmorp.megabank.tohoku.ac.jp). For autosomal recessive inheritance and compound heterozygous pattern, we used slightly relaxed filters: a CADD score of <10, and a maximum allele frequency of >2.5%.

Patient 2: The genomic DNA was extracted from a peripheral blood sample using a QIAamp Blood Mini Kit (Qiagen, Hilden, Germany). The extracted DNA was used to prepare an exome library using a TruSight One sequencing panel kit (Illumina, San Diego, CA, USA) comprising the exomes of 4,813 known disease-associated genes and containing all the reagents necessary for indexing, amplification, enrichment, and sequencing of the sample. The exome library was sequenced on a MiSeq system (illumina) in 151-cycle pair-end mode. The MiSeq Reporter v.2.5 software (Illumina) preinstalled on the MiSeq system was used to align the resulting sequence reads to a reference genome (UCSC hg19) (BWA 0.6.1-r104-tpx) and to carry out variant calling (Genome Analysis Tool Kit, GATK 1.6-22-g3ec78bd). The variant call files were imported into the VariantStudio v2.2 software (Illumina) for annotation and filtering. Only variants passing the quality PASS filter of the VariantStudio v2.2 were considered for continuation of the analysis. For variant filtering, the following variables were used: variants with a minor allele frequency of >1% in the 1000 genomes project database (April 2012 phase 1 call set) or the Exome Aggregation Consortium database (Cambridge, Massachusetts, http://exac .broadinstitute.org, accessed August 2016), and synonymous variants were excluded. All remaining frameshift insertions/deletions, stop lost, stop gained, missense, and mutations at highly conserved splice sites were investigated.

Patients 1 and 2: Finally, the candidate variant, c.1090C>T (p.Arg364Trp) in the *MFN2* gene, was confirmed by Sanger sequencing with the primer pair: 5'-CTGTGTCCCTGGCAGTGAAAAC-3' (forward) and 5'-AAATGTGTTCAGGCTCTGAGAATGG-3' (reverse).

Supplementary figure 1. Brain MRI FLAIR image of patient 2.

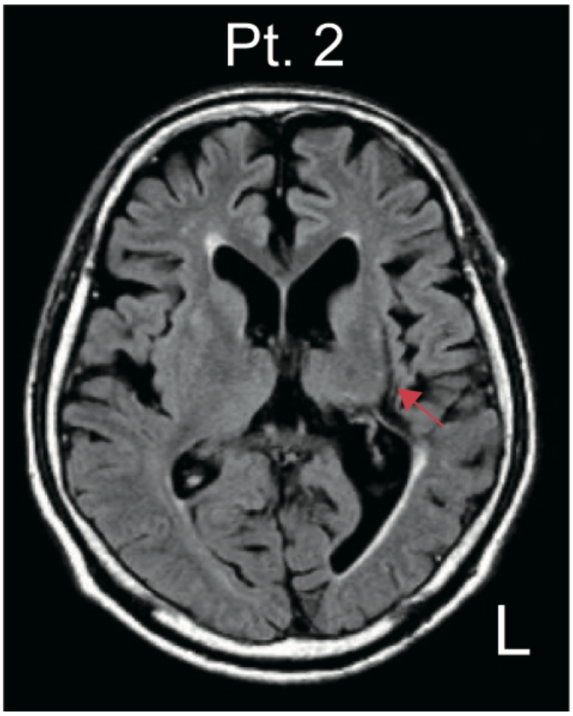

An old putaminal hemorrhage is evident in the form of a slit-like cystic cavity within the lateral side of the left putamen (*arrow*). FLAIR, fluid attenuated inversion recovery; Pt, patient; L: left side of the brain.

Supplementary figure 2. Neuropathologic findings in the cerebellum and motor cortex of patient 1.

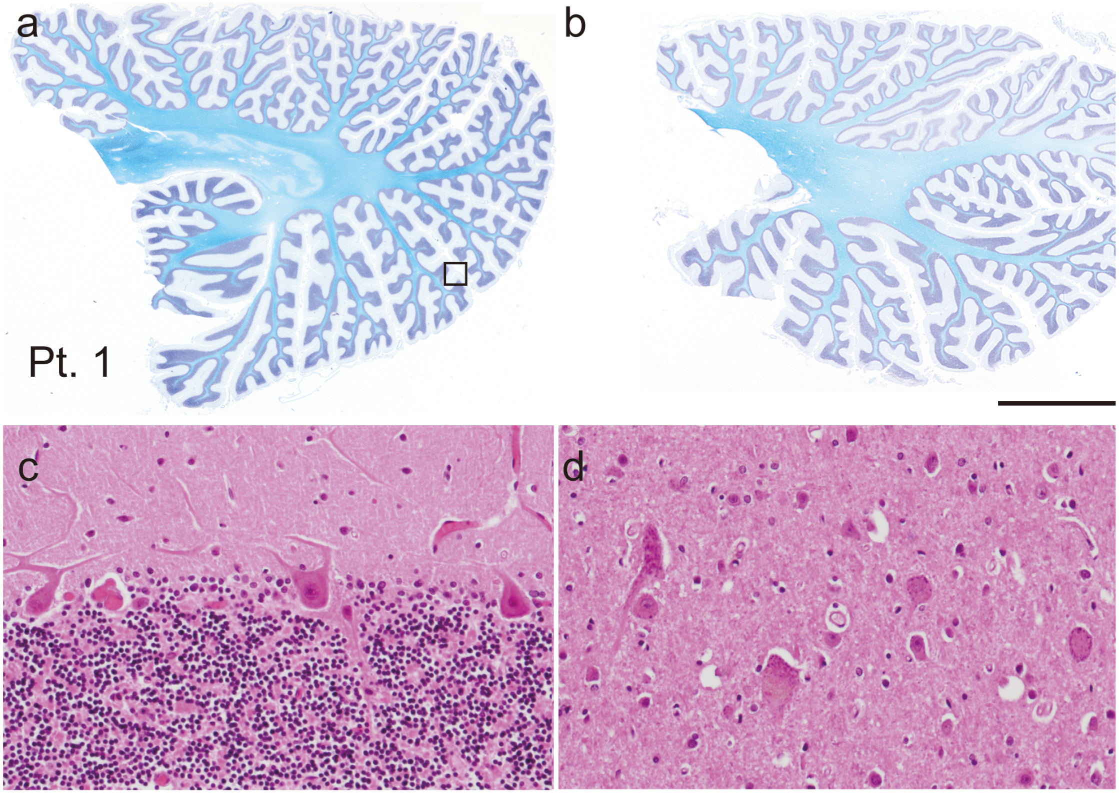

Sagittal sections of the cerebellum **(a)** at the level of the dentate nucleus and **(b)** on the lateral side. The lateral cerebellar white matter shows more pronounced myelin pallor than the median side. Klüver-Barrera (KB) staining. **(c)** Magnified image of the area enclosed by the square in **(a)**, showing a well-preserved cerebellar cortex. HE staining. (**d**) No apparent microscopic changes are evident in the primary motor cortex. HE staining. Bar in **a** = 10 mm in **a**, **b**; 100 µm in **c**, **d**.

Supplementary figure 3. Staining for ubiquitin and other antigens in spinal anterior horn cells.

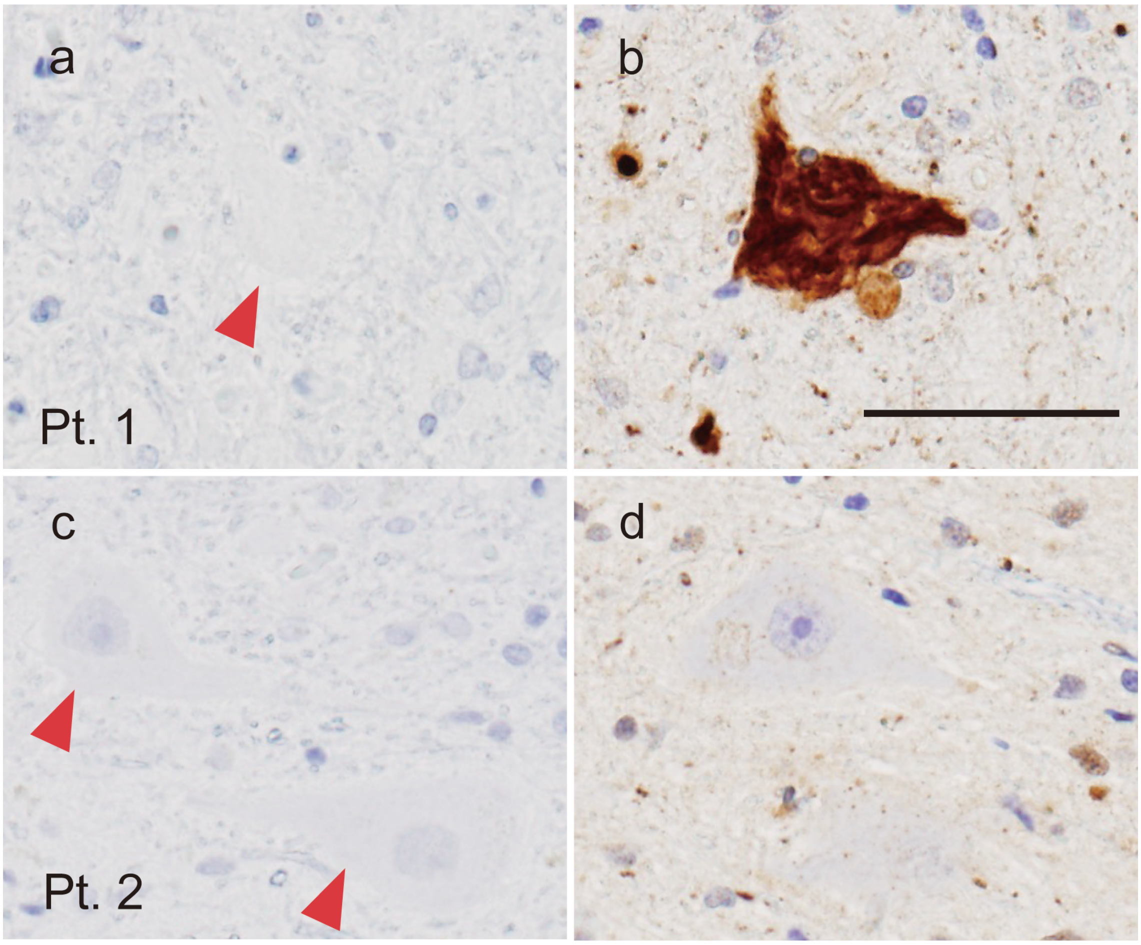

pTDP-43 **(a, c)** and ubiquitin **(b, d)** immunostaining in serial sections of the lumbar anterior horn cells in patient 1 **(a, b)** and patient 2 **(c, d)**. No pTDP43 immunopositivity is evident (*arrowheads*) in both patients (**a**, **c**). (**b**) A neuron with ubiquitin-positive structures. **(d)** No ubiquitin immunoreactivity is evident in patient 2. Bar in **b** = 50 μm in **a-d.**

Supplementary figure 4. Ultrastructure of mitochondria in the axons of the optic nerves, and proximal and distal segments of posterior nerve roots (formalin-fixed samples).

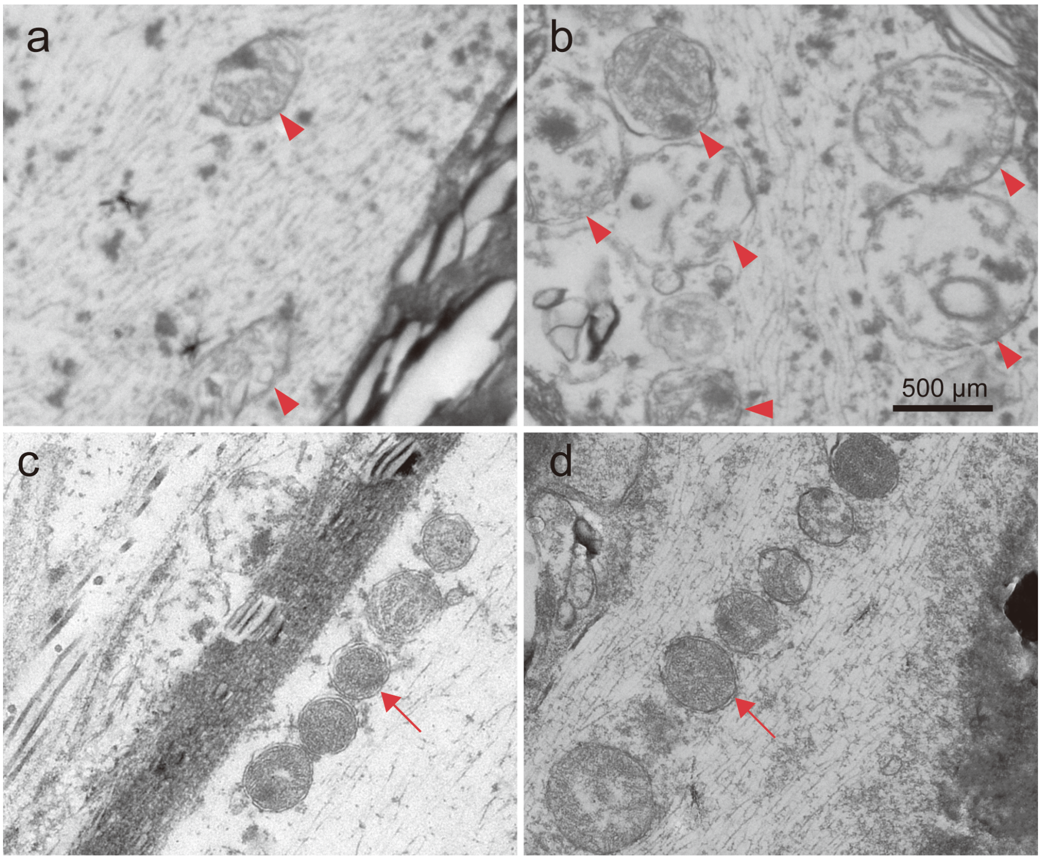

Longitudinal section of **(a, b)** the optic nerves obtained and **(c)** proximal and **(d)** distal segments of the posterior nerve roots from patient 1. In the optic nerves, **(a)** round mitochondria are scattered within the axons (*arrowheads*) and **(b)** a small number of apparently abnormal mitochondrial aggregates were also observed (*arrowheads*). Due to severe impairment of the optic nerves, observable optic nerve axons were limited. To study whether there were any differences in the ultrastructure of mitochondria within axons in the proximal and distal segments of the same nerve, both the proximal and distal segments of the posterior spinal nerve roots were assessed. **(c, d)** In the posterior nerve root, similar abnormal mitochondrial aggregates were identified in the proximal segment as well as the distal segment (*arrows*). There were no obvious differences in the ultrastructure of mitochondria within the axons of the posterior nerve roots obtained. Bar in **b** = 500 µm for **a-d**.
